# A Case of Dubin-Johnson Syndrome in Pregnancy

**DOI:** 10.7759/cureus.4048

**Published:** 2019-02-11

**Authors:** Avantika Gupta, Purnima Tiwari, Poonam Sachdeva

**Affiliations:** 1 Obstetrics & Gynecology, Jawaharlal Institute of Postgraduate Medical Education and Research (JIPMER), Puducherry, IND; 2 Obstetrics & Gynecology, Maulana Azad Medical College, New Delhi, IND

**Keywords:** dubin johnson syndrome, hyperbilirubinemia

## Abstract

Dubin-Johnson syndrome is an autosomal recessive condition characterized by recurrent episodes of jaundice and conjugated hyperbilirubinemia. It exacerbates during pregnancy and needs to be differentiated from other causes of jaundice. A 30-year-old patient presented to us with jaundice in her fourth pregnancy. She had intermittent episodes of jaundice earlier, with exacerbation in each pregnancy during the second trimester. The diagnosis of Dubin-Johnson syndrome was made on detailed evaluation along with histopathological confirmation on liver biopsy tissue. The patient was managed conservatively and had a good perinatal outcome.

## Introduction

Dubin-Johnson syndrome is a chronic, benign, intermittent jaundice with conjugated hyperbilirubinemia and impaired secretion of anion conjugates from hepatocytes into the bile. It is an autosomal recessive condition and the mutation responsible is in the gene encoding MRP-2 (multidrug resistance-associated protein-2), which transports bilirubin glucuronides across the canalicular membrane [1]. The hepatocytes show a brown pigment, which is neither iron nor bile. Pruritus is typically absent and the serum alkaline phosphatase and bile acids are within normal range. Jaundice exacerbates during pregnancy, as reported in the current case. In this case, the patient had an exacerbation of jaundice in all four pregnancies.

## Case presentation

A 30-year-old female had an onset of intermittent jaundice for the last six years. She was fourth gravida, with one live issue after a fourth-degree consanguineous marriage. She had episodes of jaundice during each pregnancy, which used to start in the late second trimester. The jaundice subsided slowly over two to three months after delivery each time. In between pregnancies, there was minimal or no jaundice. However, the patient was otherwise asymptomatic throughout these years. There was no history of any fever, pruritus, malaise, or abdominal pain. She was non-alcoholic and a non-smoker. There was no history of any blood transfusion, intravenous drug abuse, chronic drug intake, or occupational hazard.

Her pre-pregnancy bilirubin levels were in the range of 3.0-4.0 mg/dL (normal value 0.1-1.2 mg/dL) and would rise to about 11.0-12.0 mg/dL during the second trimester of each pregnancy. She typically had an isolated conjugated hyperbilirubinemia with conjugated bilirubin levels rising to as high as 8.0 to 10.0 mg/dL while the unconjugated bilirubin used to be in the range of 2.0-3.0 mg/dL. The liver function test was invariably normal. Serum transaminase levels were always less than 20 IU/L and serum bile acid levels and proteins were within normal range. The absence of pruritus, normal bile acid levels (0-10.0 µmol/L) and serum transaminases (<20 IU/L) excluded the diagnosis of intrahepatic cholestasis of pregnancy. There was no associated gestational hypertension or proteinuria. Her hemogram was also unremarkable and peripheral smear never showed any evidence of hemolysis. Thus, thrombotic microangiopathies were also ruled out in the patient. Ultrasound abdomen showed a normal liver span and echotexture. The viral markers for hepatitis were also negative in each pregnancy. These tests were done to rule out any active viral hepatitis and included anti-hepatitis A virus immunoglobulin M (IgM) antibody, hepatitis B virus antigen, anti-hepatitis B virus immunoglobulin G (IgG) and IgM antibodies, anti-hepatitis C virus IgM antibody, and anti-hepatitis E IgM antibody. Autoimmune hepatitis was also ruled out, as serum antinuclear antibodies and anti-smooth muscle antibodies were absent. The normal levels of gamma-glutamyl transferase excluded obstructive biliary disorders.

**Figure 1:**
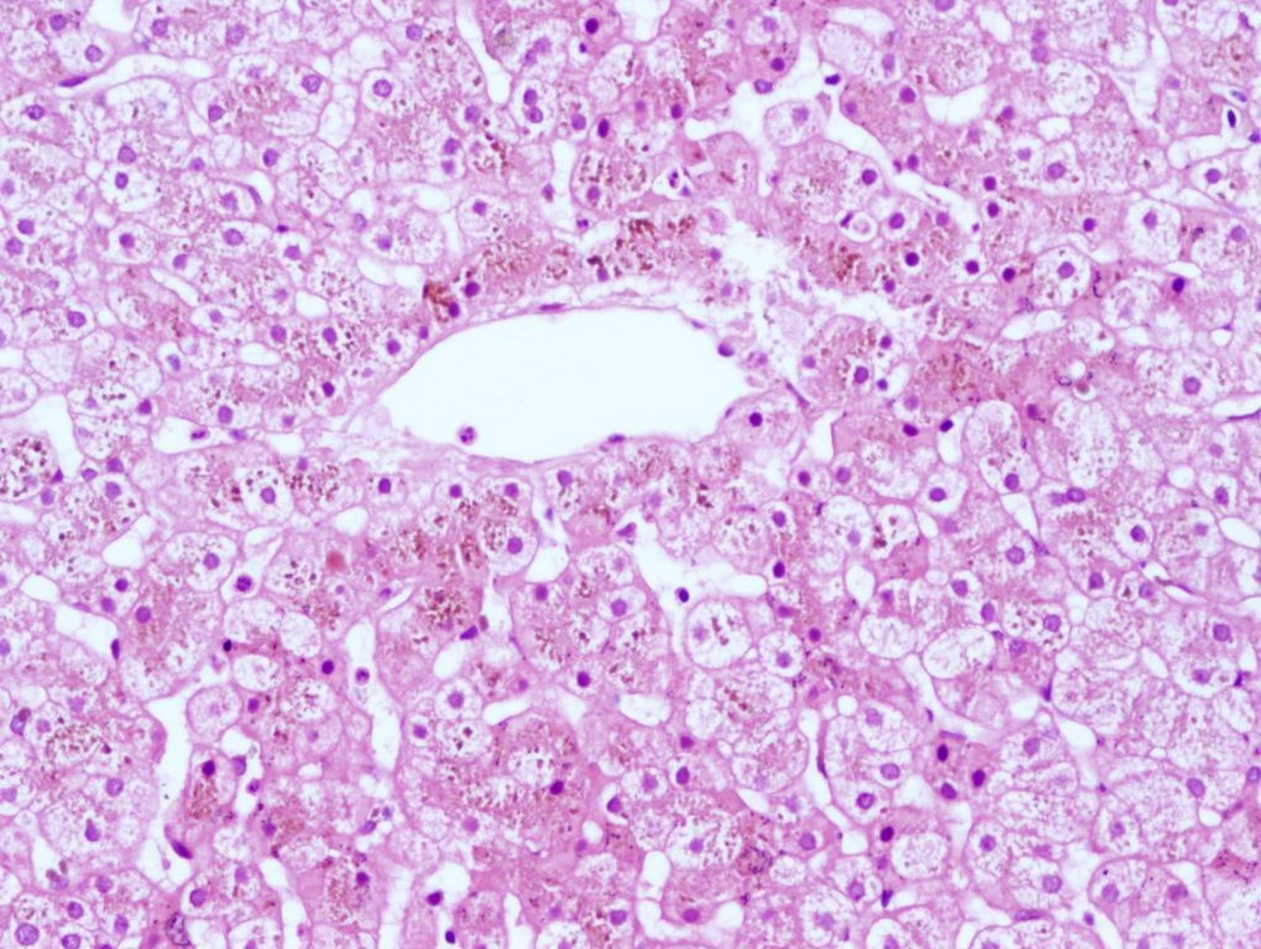
Microscopic appearance of liver tissue on hematoxylin and eosin stain (20X view) Collection of brown coarse granular intracytoplasmic pigments in perivenular hepatocytes in case of Dubin-Johnson syndrome

**Figure 2:**
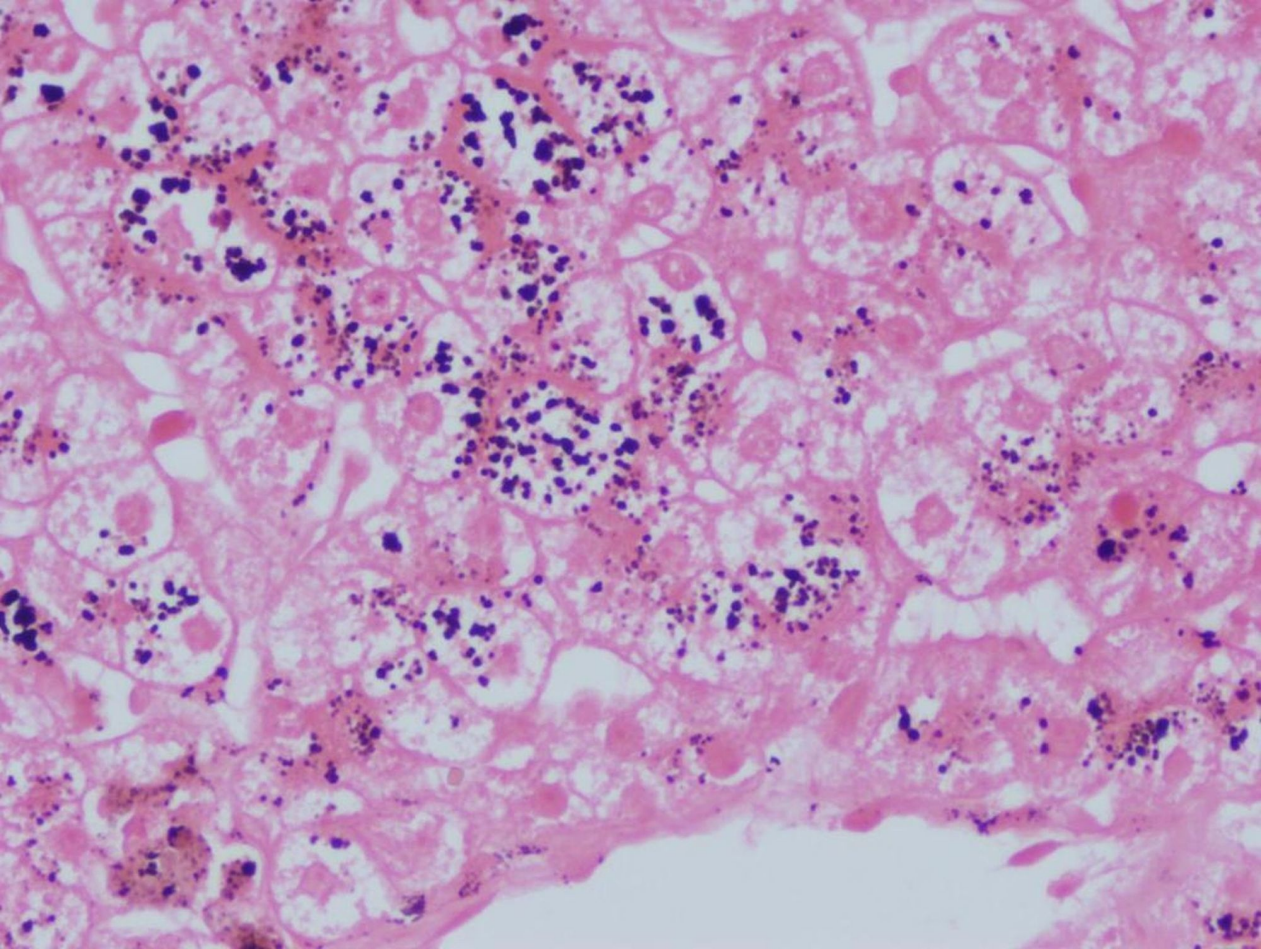
Liver tissue on Masson Fontana staining (40X view) Intracytoplasmic pigment stains black on Masson Fontana staining

Urine was positive for urobilinogen owing to conjugated hyperbilirubinemia. Total urine coproporphyrin levels were normal, however, the ratio of isomer I:III was not done due to the non-availability of the test. Since the ultimate diagnosis of Dubin-Johnson syndrome is made on liver histology, a liver biopsy was done six months after her delivery, which showed normal lobular architecture. The hepatocytes showed intracytoplasmic coarse brown pigment located maximally in the perivenular hepatocytes (Figure 1). The same pigment stained black with Masson Fontana stain (Figure 2) and was negative for periodic acid-schiff (PAS) and Perl stain. The presence of liver pigment on liver histology and the normal levels of total urine coproporphyrin levels differentiated the condition from Rotor syndrome. Genetic studies for MRP-2 gene mutation could not be done due to financial constraints. Based on the clinical picture, investigations, and liver biopsy report, a final diagnosis of Dubin-Johnson syndrome was made.

Since it is a benign condition without any complications, she was reassured of a normal course of the disease. She became pregnant one year after the diagnosis. At 30 weeks, she again presented with an exacerbation of jaundice, which was not progressive. Her serum bilirubin levels were in the range of 10.0-11.0 mg/dL with predominant conjugated hyperbilirubinemia. She went into spontaneous labor at 40 weeks and delivered a healthy male baby of 3.2 kg.

In her previous pregnancies, her second baby had a giant encephalocele and died at 15 days of life while the third baby died of severe diarrhea at the age of six months. None of them had jaundice.

## Discussion

The differential diagnosis of jaundice in pregnancy includes viral hepatitis, intrahepatic cholestasis of pregnancy, acute fatty liver of pregnancy, HELLP (hemolysis, elevated liver enzymes levels, and low platelet levels) syndrome, and, rarely, hereditary disorders like Dubin-Johnson syndrome and Rotor syndrome.

Dubin-Johnson syndrome is a rare autosomal recessive liver disorder characterized by chronic or intermittent conjugated hyperbilirubinemia (non-pruritic) and is caused by deficient, multidrug resistant-associated protein-2 [1]. In women, the disease may be discovered when jaundice appears during pregnancy or with the use of oral contraceptive pills. Our patient had an exacerbation of jaundice in each subsequent pregnancy. No treatment is required and the fetal outcome is usually not affected [2]. There is a defect in the excretion of radiographic dye and a non-bile salt organic anion such as bromosulfophthalein dye and urobilinogen. Hence, there is a delayed rise of plasma levels of bromosulfophthalein after the injection of the dye due to the reflux of conjugated anion from hepatocytes as well as the presence of excess urobilinogen in urine [3]. Liver function tests, including serum bile acid levels, are normal. Serum bilirubin fluctuates usually from 2-5 mg/dL and over 50% of the total serum bilirubin is conjugated and excreted in urine. In patients with isolated conjugated hyperbilirubinemia with otherwise normal liver function tests, the diagnosis of Dubin-Johnson syndrome can be confirmed by demonstrating an increase in the ratio of urine coproporphyrin I to III isomer value; type I makes up to 80% rather than the usual 25% of the total urinary coproporphyrin levels in these patients [4]. We avoided the classical bromosulfophthalein test because of the potentially severe side effects.

The microscopic appearance of the liver is normal, except that coarse, iron-free, dark brown granules accumulate in hepatocytes and Kupffer cells, primarily in the centrilobular zone. Accumulation of this pigment causes the liver to be grossly pigmented and blackish in color [5]. Since hepatocytes do not synthesize melanin, it has been suggested that the pigment reflects the auto-oxidation of anionic metabolites, particularly epinephrine. Rotor syndrome is clinically similar to Dubin-Johnson syndrome except for the appearance of liver cells, which are not pigmented in Rotor syndrome [5]. The intracellular pigment present in Dubin-Johnson syndrome is coarse and brown in color. It stains positive by Masson Fontana stain and negative for PAS and Perl or Gomori’s iron stain [6]. Melanin pigment should be differentiated from lipofuscin and hemosiderin deposits. Lipofuscin forms brown, fine granules, usually perinuclear located, representing undigested material from lipid peroxidation, and is associated with aging. It is positive by PAS stain and negative by Masson Fontana stain and doesn’t disappear after bleaching. Hemosiderin is an intracellular storage form of iron and appears as golden yellow-brown intracellular or extracellular pigment. It stains positive by Gomori, Perl, or Lillie’s iron stain [6].

Viral hepatitis is the most common cause of jaundice in pregnancy [7]. History of arthralgia and myalgias predating jaundice suggests hepatitis. Patients with acute viral hepatitis typically have aminotransferase levels greater than 500 IU/L with alanine aminotransferase (ALT) greater than or equal to the aspartate aminotransferase (AST) [8].

Intrahepatic cholestasis of pregnancy is second only to viral hepatitis as a cause of jaundice in pregnancy. Pruritus (without rash) is the chief complaint in intrahepatic cholestasis. Jaundice may occur and typically develops one to four weeks after the onset of symptoms. Pruritus usually disappears within 24-48 hours of delivery [9]. The recurrence risk in subsequent pregnancies is 40%-60% [10]. Serum levels of bile acids are elevated from 10 to 100 fold in patients with intrahepatic cholestasis of pregnancy. Hyperbilirubinemia results from the retention of conjugated pigment, but total plasma concentration rarely exceeds 4-5 mg/dL. Serum transaminases activities are normal to moderately elevated and seldom exceed 250 IU/L [11]. Liver biopsy shows mild cholestasis with intracellular bile pigments and canalicular bile plugging without necrosis [5].

Acute fatty liver of pregnancy is an autosomal recessive condition in which there is a mitochondrial abnormality of fatty acid oxidation. It typically occurs in the third trimester. There is hypoglycemia, acute liver, and renal failure with encephalopathy. Laboratory findings are hyperbilirubinemia of less than 10 mg/dL, increased alkaline phosphatase, prolonged clotting time and prothrombin time, and modest elevations of serum transaminases [12]. Liver biopsy shows fatty changes [7]. HELLP syndrome is characterized by severe pre-eclampsia, hemolysis, elevated serum transaminases, and thrombocytopenia [7].

Multidrug resistance protein causes the transfer of bilirubin to bile canaliculi from hepatocyte allowing the transport of glucuronide and glutathione conjugates back into the blood. The absence of multidrug resistance protein-2 from the hepatocyte canalicular membrane is the basis of this syndrome. Various mutations in the MRP-2 gene can lead to Dubin-Johnson syndrome [13]. In a single large case series of 20 cases of Dubin-Johnson syndrome, Rastogi et al. [14] highlighted clinical and pathological findings. All patients had conjugated hyperbilirubinemia with a mean value of total serum bilirubin of 4.4 mg/dL. Liver biopsies revealed the presence of coarse granular brown pigment in the cytoplasm of hepatocytes, more concentrated in the pericanalicular region and more prominent in centrilobular hepatocytes.

Dubin-Johnson syndrome is a rare clinical entity. Its definitive diagnosis is made on the histopathology of liver biopsy tissue, although a genetic mutational analysis of genes encoding MRP-2 have also been described in the literature in order to make a more accurate diagnosis of the causative mutation [15]. Management is conservative unless other co-morbidities arise. This is one of the few case reports of pregnancy management in a patient with Dubin-Johnson syndrome and emphasizes that fetal outcome is usually not affected when proper supportive management is provided to the mother. The patient should be counseled regarding the benign nature of the disease and its possible exacerbation during future pregnancies, oral contraceptive pill use, and during an intercurrent illness.

## Conclusions

To conclude, the possibility of Dubin-Johnson syndrome should be considered when evaluating a patient with asymptomatic conjugated hyperbilirubinemia. It needs to be differentiated from various other conditions leading to hyperbilirubinemia, especially in pregnancy, in order to prognosticate the patient and to strategize further management.
